# A new strategy of using satellite RNA to control viral plant diseases: post‐inoculation with satellite RNA attenuates symptoms derived from pre‐infection with its helper virus

**DOI:** 10.1111/pbi.13145

**Published:** 2019-05-15

**Authors:** Xinran Cao, Shanshan Liu, Chengming Yu, Xiangdong Li, Xuefeng Yuan

**Affiliations:** ^1^ Department of Plant Pathology College of Plant Protection Shandong Agricultural University Shandong Province Key Laboratory of Agricultural Microbiology Tai'an China; ^2^ Yantai Agricultural Technology Extension Center Yantai China

**Keywords:** satellite RNA, viral plant diseases, post‐inoculation, *Cucumber mosaic virus*

Satellite RNA (satRNA) is the supernumerary RNA of some viruses. It typically shares no nucleotide homology with its helper virus and depends on its helper virus for replication and encapsidation (Simon *et al*., 2004). The presence of satRNA sometimes interferes with the replication and symptom expression of its helper virus. Satellite RNA can have neutral, attenuated or intensified effects on the virulence of its helper virus owing to complicated interactions among satRNA variants, helper viruses and host genotypes (Cillo *et al*., 2004; Simon *et al*., 2004). Attenuated satRNAs have been used as biocontrol agents of plant diseases caused by their helper viruses in two ways. The first is cross‐protection, in which a combination of satRNA and its helper virus is inoculated onto target plants in advance to prevent them from further pathogenicity of helper virus (Sayama *et al*., 2001; Tian *et al*., 1985; Yoshida *et al*., 1985). Secondly, satRNA‐transgenic plants have been created which convey phenotypes resistant against the virulence of the helper virus (Baulcombe *et al*., 1986; Cillo *et al*., 2004; Gerlach *et al*., 1987). The mechanisms by which satRNA triggers resistance to helper viruses include competition for viral replicase, gene silencing and possibly other immune actions of the host (Simon *et al*., 2004; Ziebell and Carr, 2010). Current methods of using satRNA as a biocontrol agent are preventive strategies that must be applied before infection of the target viruses. In this study, the effects of post‐inoculation with satRNA on symptoms derived from pre‐infection by its helper virus (*Cucumber mosaic virus*, CMV) were tested and screened. Post‐inoculation with satRNA could be a new way to control diseased plants pre‐infected by helper viruses and could form a new therapeutic strategy to combat plant viruses using attenuation satRNAs.

Liu *et al*. (2018) identified a new satRNA isolate of CMV from tobacco in China and termed it satCMV TA‐tb (Accession No. MF142365). The isolate is 383 nt in length. They found that co‐inoculation with satCMV TA‐tb and CMV_Fny_ remarkably inhibited symptoms caused by CMV_Fny_. CMV_Fny_ infection caused severe dwarfing of the whole plant along with severe crinkling of the leaves in *Nicotiana benthamiana*, but when plants were co‐inoculated with satCMV TA‐tb and CMV_Fny_ symptoms were reduced to slight stunting. In the present study, to identify the effects of post‐inoculation with satRNA on pre‐infection with CMV, *N. benthamiana* plants infected with CMV_Fny_ were post‐inoculated with satCMV TA‐tb at 3, 5, 7 or 10 dpi (Figure 1a). The inoculation method used for both CMV_Fny_ and satCMV TA‐tb was as follows: fresh single colonies of Agrobacterium *GV3101* with plasmids containing CMV_Fny_ RNA1, CMV_Fny_ RNA2, CMV_Fny_ RNA3 or satCMV TA‐tb were incubated in LB with 100 μg/mL rifamycin and 50 μg/mL kanamycin for 48 h at 28 °C. Fifty micro liter overnight‐incubated LB was then added to 5 mL LB with 100 μg/mL rifamycin, 50 μg/mL kanamycin mL, 10 mmol/L 2‐(N‐Morpholino) ethanesulfonic acid (MES) and 20 μmol/L Acetosyringone (AS). This was followed by incubation at 28 °C until the logarithmic growth phase. After centrifugation, the pellets were resuspended in an agro‐infiltration buffer (10 mmol/L MgCl_2_, 10 mmol/L MES, 150 μmol/L AS) to adjust OD_600_ to 1.5 for CMV_Fny_ RNA1, CMV_Fny_ RNA2 or CMV_Fny_ RNA3 or to 0.5 for satCMV TA‐tb, which was then incubated for 3 h at 28 °C. To agro‐infiltrate CMV_Fny_, equal volumes of resuspended Agrobacterium *GV3101* containing CMV_Fny_ RNA1, CMV_Fny_ RNA2 or CMV_Fny_ RNA3 were mixed and agro‐infiltrated onto the third and fourth leaves of plants using a blunt end syringe. At 3, 5, 7 or 10 dpi, Agrobacterium *GV3101* containing satCMV TA‐tb was agro‐infiltrated onto the fifth and sixth leaves of plants using a blunt end syringe. At 24 days post‐CMV_Fny_ infection, *N. benthamiana* presented severe dwarfing along with severe crinkling of leaves (Figure 1b,c). Post‐inoculation with satCMV TA‐tb at 3 or 5 dpi remarkably ameliorated symptoms on the upper part of the inoculated leaves, which were slightly dwarfed and explanate (Figure 1b,c). Post‐inoculation with satCMV TA‐tb at 7 dpi did not ameliorate the dwarfing, but reduced flatness of the upper leaves (Figure 1b,c). Post‐inoculation with satCMV TA‐tb at 10 dpi produced a neutral effect on symptoms caused by CMV infection (Figure 1b,c). Taken together, these results demonstrate that post‐inoculation of satCMV TA‐tb on upper younger leaves at suitable time points can effectively control disease in *N. benthamiana* pre‐infected by CMV_Fny_.

**Figure 1:**
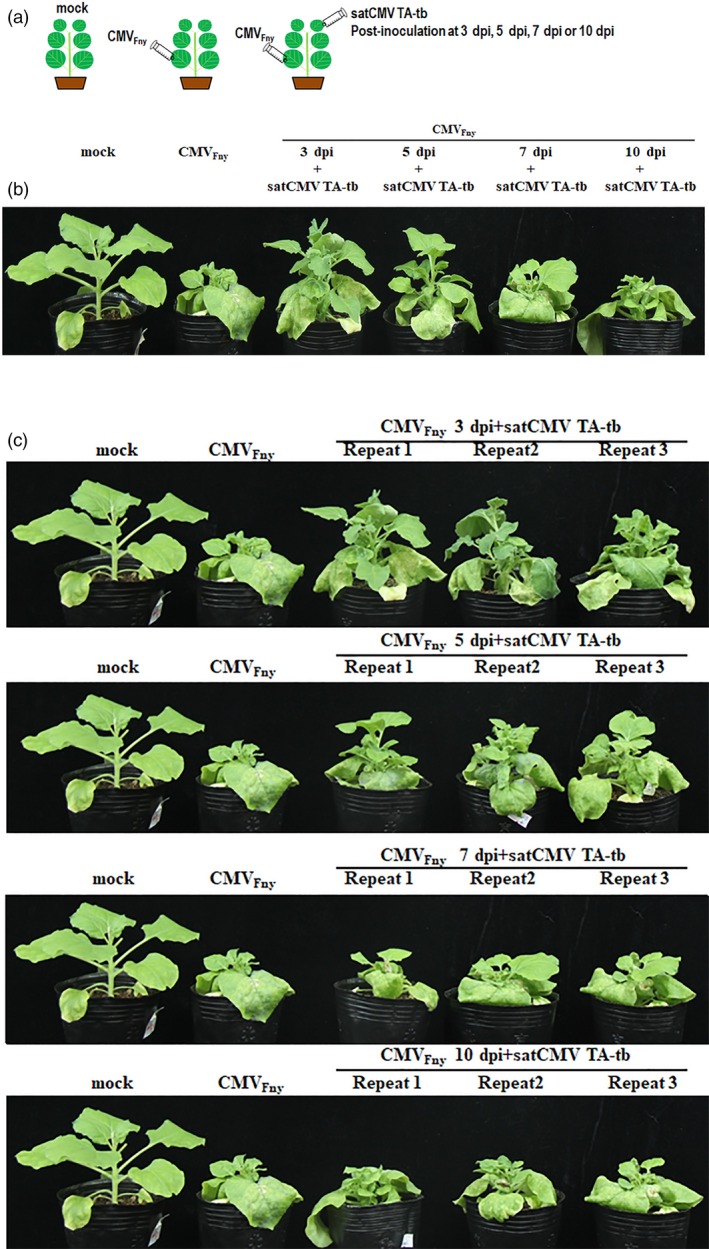
Effect of post‐inoculation of satCMV TA‐tb on *N. benthamiana* pre‐infected with CMV_F__ny_. (a) Diagram of post‐inoculation of satCMV TA‐tb. (b) Effect of post‐inoculation of satCMV TA‐tb at different time point on *N. benthamiana* infected with CMV_F__ny_ at 24 dpi. (c) Repeated analysis of the effects of post‐inoculation of satCMV TA‐tb at different time points on *N. benthamiana* infected with CMV_F__ny_ at 24 dpi.

Viruses are responsible for about 50% of all plant diseases. All major crops have been suffering economic losses as a result of viruses (Ziebell and Carr, 2010). To protect plants from viruses, several strategies have been generated such as producing virus‐free propagates, inducing cross‐protection, preventing the spread of viruses via vectors, and developing virus‐resistant plants through conventional breeding, transgenesis or gene editing (Ziebell and Carr, 2010). In addition to inherent virus‐resistant breeding, other effective strategies to control of virus diseases must be applied before viral infections occur or be used to block the dissemination of virus diseases. However, before now, there had been little implementation of or focus on therapies to treat diseased plants pre‐infected with viruses. This study demonstrates that post‐inoculation of satRNA on younger leaves could be a considerable and potentially powerful way to treat diseased plants to a certain extent, owing to the abundant resource of satRNAs corresponding to at least 26 plant viruses from six groups (Fritsch and Mayo, 1988). In addition, post‐inoculation of satRNA presents more valuable potential in control of the diseased perennial in which virus is gradually accumulated year by year. Current applied strategy such as cross‐protection had no chance to be applied on the diseased perennial due to the pre‐existence of viruses in plants.

In terms of creating a potential therapeutic strategy, it is important to determine the window phase for post‐inoculation with satRNA. In this study, 5 days was the limitation of the window phase for post‐inoculation with satCMV TA‐tb that produced a therapeutic effect on plants pre‐infected with CMV. However, the virus titre used to infect plants in this study is much higher than that in the field. The window phase for post‐inoculation with satRNA could be longer than 5 days in the field owing to interactions among satRNA variants, helper viruses and host genotypes. Using supersensitive detection methods on viruses such as RT‐PCR, immune‐capture RT‐PCR, PCR‐ELISA and LAMP, viruses can be detected in the early stages of infection in the field, which could ensure enough time for post‐inoculation with the corresponding attenuation satRNA via agro‐infiltration using a blunt end syringe or a compression sprayer. Post‐inoculation with satRNA is sure to play an essential role in the control of diseased plants owing to its many advantages. This strategy is simple and convenient to apply, which can partially compensate for the shortcomings of other strategies used to control diseased plants pre‐infected with viruses. This strategy of post‐inoculation with satRNA can not only control diseased plants pre‐infected by its helper virus as shown in this study, but could also prevent new infections by its helper virus via cross‐protection after post‐inoculated satRNA interacts with pre‐infected helper virus. Satellite RNA, a feature that distinguishes plant viruses from animal viruses, is guaranteed to become a key player in the control of viral plant diseases.

## Author contributions

X.Y. designed the experiments and wrote the manuscript. X.L. and X.Y. supervised the research. X.C., S.L. and C.Y. contributed to the clone of satellite RNA, vector construction and agro‐infiltration. All authors discussed the data.

## Conflict of interest

The authors declare that they have no competing financial interests to disclose.
